# Antitumor properties of two traditional aromatic rice genotypes (*Kalijira and Chinigura*)

**Published:** 2014

**Authors:** Mohammad Abdul Mannan, Tushar Chandra Sarker, Ahmad Humayan Kabir, Mostafizur Rahman, Mohammad Firoz Alam

**Affiliations:** 1 *Plant Biotechnology and Microbiology Laboratory, Department of Botany, University of Rajshahi, Rajshahi 6205, Bangladesh*

**Keywords:** *Antitumor activity*, *Traditional rice*, *Tumor inhibition*, *Unpolished grain*

## Abstract

**Objective: **Methanol extract of bran and unpolished grain of two traditional aromatic rice genotypes viz. Kalijira and Chinigura were assayed for their activity on the growth and initiation of crown-gall tumors on potato disks.

**Materials and Methods:** Three *Agrobacterium tumefaciens* (*A. tumefaciens)* strain AtSl0105, AtTa0112, and AtAc0114 were used as the tumor forming agent. Collected rice was separated to bran and unpolished grain by different milling processes and made into fine powder before extracting using methanol. Antitumor assay of plant extracts was performed according to standard potato disc bioassay. Disc diffusion assay (Kirby-Bauer Method) was used to screen *A. tumefaciens* sensitivity test.

**Results:** The results demonstrated a high correlation between the ability of aromatic rice to inhibit the initiation and growth of crown-gall tumors on potato disks. Maximum tumor inhibitions were observed against the strain AtSl0105 by Kalijira bran (73.91%) and Chinigura bran (69.56%). Both unpolished grains showed significant effect (Kalijira 57.43%, Chinigura 55.53%) to inhibit the tumor.

**Conclusion: **It can be concluded that aromatic rice (Kalijira and Chinigura) might be a potential source of antitumor agent that can be used for further drug development for tumor treatment.

## Introduction

A localized proliferation of tissue forming a swelling or outgrowth, commonly with a characteristic shape and unlike any organ of the normal structure is known as tumor. Plant tumors are also known as Galls. Plant galls or crown galls usually form in response to the action of a pathogen or a pest (Holliday, 1989 ▶). Crown-gall is a neoplastic disease of plants caused by *A*. *tumefaciens* following by the transfer and expression of its special type of DNA segment (T-DNA) in the plant genome through type IV secretion system (T4SS) (Zupan et al., 2000 ▶). T4SS is also used by other pathogenic bacteria to deliver macromolecules detrimental to the host such as plant, animal and human (Cascales and Christie, 2003 ▶). Among those, *Bartonella henselae *(Kempf et al., 2002 ▶) and *Helicobacter pylori *(Raderer et al., 1998 ▶) are two tumor causing bacteria in human and they share a similar pathogenicity strategy to plant pathogen *A. tumefaciens *(Zhu et al., 2000 ▶). The above mentioned relation and previous studies have documented the similarities between crown-gall tumors and animal cancers especially the correlation between antileukemic activity and inhibition of crown-gall tumor formation on potato discs by some medicinal herbs (Anderson et al., 1988 ▶). Potato disc is a useful test for monitoring the inhibition of crown-gall tumors (McLaughlin, 1991 ▶). The inhibition of crown-gall tumor initiation on potato discs showed good agreement with compounds and plant extracts known to be active in the 3PS (*in vivo*, mouse leukemia) antitumor assay (Galsky et al., 1980 ▶; Islam et al., 2009 ▶; Sarker et al., 2011 ▶) and also the inhibition of tumors growth agrees well with 3PS activity (Galsky et al., 1981 ▶).

Cancer remains the largest cause of mortality in the world, claiming over 6 million lives each year (Abdullaev, 2002). Anticancer drug therapies induce apoptosis in cancer cells but are mostly toxic, immune-suppressive, mutagenic, and carcinogenic (Sanderson et al., 1996 ▶; Santin et al., 2000 ▶). In contrast, natural drug have attracted more and more interests because of their safety and wide distribution properties in recent years (Lewis, 1993 ▶; Hatate et al., 1990 ▶). Consumption of plant-based foods, including fruits, vegetables and whole grains, cereals, and nuts plays a pivotal role in disease prevention and health promotion. It is widely recognized that dietary ingredients have a dual role in relation to some human diseases. They can contribute to both the causes and prevention of diseases such as cancer and atherosclerosis as well as the aging process by providing dietary fiber, proteins, energy, minerals, vitamins, and antioxidants required for human health (Nam et al., 2005). Therefore, a great deal of recent research has been focused on the development of new bioactive agents from cereals (Wenzig et al., 2005 ▶; Chung et al., 2006 ▶; Saikia and Deka, 2011 ▶). In contrast, the staple components of the human diet rice have received less attention as sources of cancer chemopreventive substances. 

It is remarkable that rice possesses special dietary importance in Asia, where the incidence of breast and colon cancer is markedly lower than in the Western world (WCRF and AICR, 1997 ▶). 

Witte et al. (1996) ▶ suggest that the potential protective effects of grains on polyps might be related to the presence of dietary constituents other than fiber or antioxidants in these foods. Scientists report that rice constituents counteract chemical-induced mutagenicity (Botting et al., 1999 ▶), tumor promotion (Yasukawa et al., 1998 ▶), carcinogenicity (Aoe et al., 1993 ▶), and establish neoplastic growth in rodents (Koide et al., 1996 ▶). Infection with *H. pylori* is the most important risk factor for gastric cancer and positive association exists between *H. pylori* and pancreatic cancer (Raderer et al., 1998 ▶). Ishizone et al. (2007) ▶ and Kawakami et al. (2006) ▶ showed that rice-fluid does show an antibiotic effect on *H. pylori* and an anti-inflammatory effect on the *H. pylori* associated gastritis. Despite the importance of rice genotypes on antitumor or therapeutic activities, no extensive studied has been performed on Bangladeshi aromatic rice.

Given the potentiality of aromatic rice as an antitumor compound, the aim of this study was to study the activity of two aromatic rice genotypes viz. Kalijira and Chinigura on the growth and initiation of crown-gall tumors on potato disks.

## Materials and Methods

**Plant materials**

Two Bangladeshi traditional aromatic rice genotypes, viz. Kalijira and Chinigura, were collected from farmers of Rajshahi region, Rajshahi 6210, Bangladesh and identified by taxonomist. Two parts of individual rice (bran and unpolished grain) were used as plant materials. 

**Preparation of extracts**

The extraction procedure was performed according to Ahmad and Beg, 2001 with some modifications. Collected rice was separated to bran and unpolished grain by different milling process and made into fine powder. About 50 g fine powder was dipped into 250 ml methanol and left for 7 days with occasional shaking. Further, tetron cloth and Whatman No. 1 filter paper was used for filtration. Filtrates were taken into glass beaker for solvent evaporation (methanol). For quick evaporation of the extra solvent from the extract, water bath (4 holes analogue, Thermostatic water bath, China) was used under 60 °C and stored at 4 °C (Akueshi et al., 2002 ▶).

 Standard formula was used to calculate yield performance of the extract as described by Ekwenye and Elegalam, 2005 ▶. Particular concentrations (10 ppm, 100 ppm, and 1,000 ppm; Note: 1 ppm = 1 mgl^-1^) of the plant extracts) of the plant extracts were prepared.

**Antitumor potato disc bioassay**

Antitumor assay of plant extracts was performed according to standard potato disc bioassay (Hussain et al., 2007 ▶). *A. tumefaciens *strains (AtSl0105, AtTa0112, and AtAc0114) were cultured on Luria-Bertani (LB) agar and then transferred to LB broth and incubated for 48 hours. Six to seven loops of broth cultures were transferred into test tube containing 10 ml phosphate buffer (PBS; pH 7.2). The following proportion was used for antitumor activity test: 600 µl test extract+150 µl sterilized distilled water (SDW) +750 µl *A. tumefaciens *in PBS. Camptothecin (30 ppm) was used as positive control replacing test extracts. Red skinned potatoes (*Solanum tuberosum *L.) were collected from local market and thoroughly washed with tap and distilled water. 

Surface sterilization of samples was performed using 0.1% HgCl_2_ solution. Potato tubers were cut into 8 mm diameter in size cylindrical pieces using cork borer and transferred into SDW containing conical flask. After washing, the cylindrical segments were cut into 5×8 mm discs and placed onto agar (15gl^-1^) plates (10 discs per plate). After that, 50 µl of appropriate inoculums were placed on the surface of each potato disc. The peti-plates were sealed with parafilm and incubated at room temperature at 27-30 °C. After 21 days, discs were stained with Lugol’s solutions (10% KI, 5% I_2_) and tumors were counted under a stereo microscope. The experiment was carried out in sterilized conditions and repeated three times. Percent inhibition of tumors was calculated as described by McLaughlin and Rogers (1998) ▶. More than 20% tumor inhibition is considered significant (Ferrigni et al., 1982 ▶). 

**Sensitivity test of** ***A. tumefaciens*** **(as a partial assay)**

Disc diffusion assay (Kirby-Bauer Method) was performed for screening *A. tumefaciens *sensitivity (Barry, 1980 ▶). Methanol was used as negative control and Kanamycin (30 μg ml^-1^), Cefuroxime (30 μg ml^-1^), Tetracycline (30 μg ml^-1^), and Rifampicin (10 μm ml^-1^) were used as positive controls. Discs (Whatman No. 1 filter paper) were impregnated with 10 µl of extracts (250 mg ml^-1 ^= 250,000 ppm), antibiotics and methanol followed by air dried, and then placed on seeded LB agar plates. About 20 µl standard bacterial cultures (48 hours incubated) were used for spreading LB agar plates. Plates were then incubated at 28-30 °C for 24 hours. The sensitivity test was evaluated by the measurement of inhibition zone’s diameter (mm) against *A. tumefaciens *strain (AtSl0105, AtTa0112, and AtAc0114). Each assay was carried out in triplicates.

## Results

**Sensitivity test of** ***A. tumefaciens*** **(as a partial assay)**

Before antitumor activity test, antibacterial assay was performed against one *A. tumefaciens* strains to check their viability against plant extracts. Among the treatments, four extracts of the two rice lines and three antibiotics showed no effect on the viability of *A. tumefaciens* strains viz. AtSl0105, AtTa0112, and AtAc0114. No inhibition zone was observed for plant’s extract as well as for antibiotics. Tumor inhibition was only observed for the plant extracts and not for the other factors.

**Antitumor potato disc bioassay**

*Effect of methanol extracts of Kalijira bran on crown-gall tumors produced by A. tumefaciens on potato discs*

**Figure 1:**
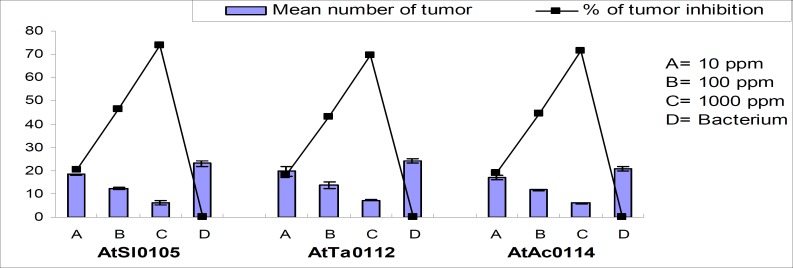
Effect of methanolic extracts of Kalijira bran on crown-gall tumor produced by *A. tumefaciens *on potato discs.

**Figure 2:**
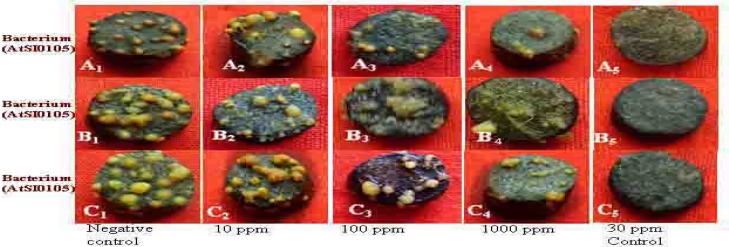
Photographs showing gradual tumor inhibition by the methanol extract of Kalijira bran on potato discs in a concentration- as well as strain-dependent manner. Data were compared with control. A_1_, B_1_, and C_1_ as negative control and A_2_, B_2_, and C_2_ as 10 ppm, A_3_, B_3_, and C_3 _as 100 ppm, A_4_, B_4_, and C_4_ as 1,000 ppm plant extract and A_5_, B_5_, and C_5_ as 30 ppm Camptothecin (positive control).

It was found that methanol extract of Kalijira bran significantly reduced tumor formation in a concentration-dependent manner across the strains (Table 1). Significant tumor inhibition was observed at 10, 100, and 1,000 ppm plant extracts compared with the negative control in all the strains. Maximum 73.91%, 69.44%, and 71.43% and minimum 20.29%, 18.06%, and 19.05% tumor inhibition were recorded for AtSl0105, AtTa0112, and AtAc0114 *Agrobacterium *strains, respectively (Figures 1 and 2). Camptothecin served as a positive control for all experiments and 100% tumor inhibition was observed. 

**Table 1:** Mean values for antitumor activity of methanol extract of Kalijira rice bran

**Variable**	**Mean number of tumor** **	**Variable**	**Mean number of tumors** **
**Strains**		Negative control	22.66 A
**AtSI0105**	14.91 A	10 ppm	18.33 B
**AtTa0112**	16.16 B	100 ppm	12.55 C
**AtAc0114**	13.91 C	1,000 ppm	06.44 D
**LSD value**	0.466	LSD value	0.539

** = significant at 5% level; ns = not significant.

*Effect of methanol extracts of Kalijira rice grain on crown-gall tumors produced by A. tumefaciens on potato discs*

**Figure 3:**
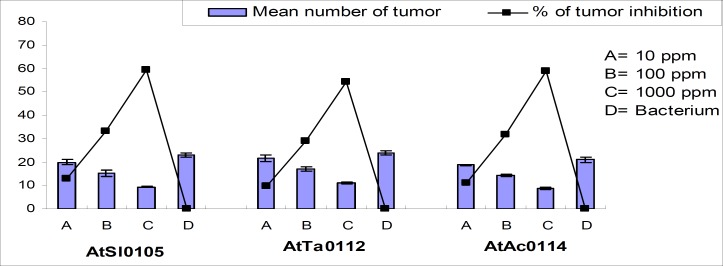
Effect of methanol extract of Kalijira rice grain on crown-gall tumor produced by *A. tumefaciens *on potato discs.

**Figure 4:**
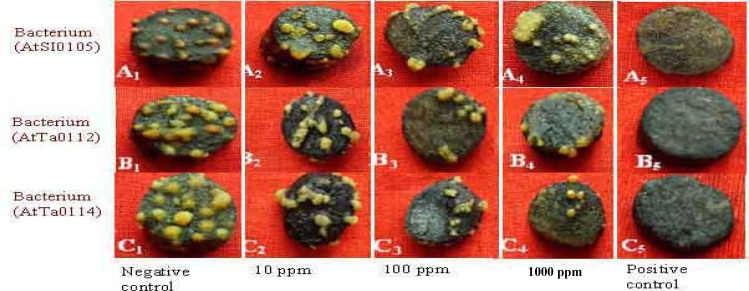
Photographs showing gradual tumor inhibition by the methanol extract of Kalijira grain on potato discs in a concentration as well as strain dependent manner. Data were compared with control. A_1_, B_1_, and C_1_ as negative control and A_2_, B_2_, and C_2_ as 10 ppm, A_3_, B_3_, and C_3 _as 100 ppm, A_4_, B_4_, and C_4_ as 1,000 ppm plant extract and A_5_, B_5_, and C_5_ as 30 ppm Camptothecin (positive control).

The methanol extract of Kalijira rice grain also significantly reduced tumor formation in a concentration-dependent manner across the strains (Table 2). Significant tumor inhibition was observed at 10, 100, and 1,000 ppm plant extracts compared with the negative control in all the strains. Maximum 59.42%, 54.17%, and 58.73% and minimum 13.04%, 9.72%, and 11.11% tumor inhibition were recorded for AtSl0105, AtTa0112, and AtAc0114 *Agrobacterium *strains, respectively (Figures 3 and 4).

*Effect of methanol extracts of Chinigura rice bran on crown-gall tumors produced by A. tumefaciens on potato discs*

**Figure 5:**
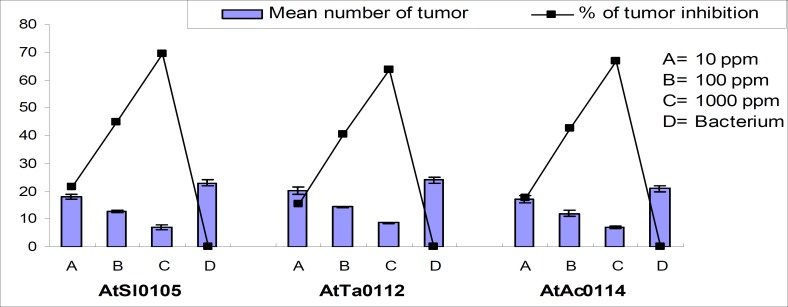
Effect of methanol extract of Chinigura rice bran on crown-gall tumor produced by *A. tumefaciens *on potato discs.

**Figure 6:**
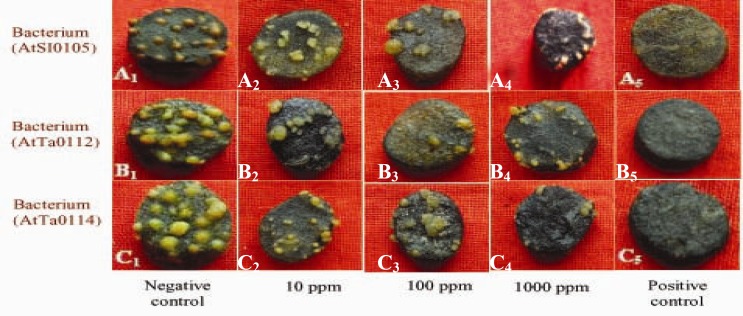
Photographs showing gradual tumor inhibition by the methanol extract of Chinigura rice bran on potato discs in a concentration as well as strain dependent manner. Data were compared with control. A_1_, B_1_, and C_1_ as negative control and A_2_, B_2_, and C_2_ as 10 ppm, A_3_, B_3_, and C_3 _as 100 ppm, A_4_, B_4_, and C_4_ as 1,000 ppm plant extract and A_5_, B_5_, and C_5_ as 30 ppm Camptothecin (positive control).

Significant tumor inhibition was observed in a concentration-dependent manner across the strains (Table 3). Maximum 69.57%, 63.89%, and 66.67% and minimum 21.74%, 15.28%, and 17.46% tumor inhibition were recorded for AtSl0105, AtTa0112, and AtAc0114 *Agrobacterium *strains, respectively for the concentration 10, 1,00, and 1,000 ppm, respectively (Figure 5 and 6).

**Table 2:** Mean values for antitumor activity of methanol extract of Kalijira rice grain

**Variable**	**Mean number of tumor** **	**Variable**	**Mean number of tumors** **
**Strains**		Negative control	22.66 A
**AtSI0105**	16.91 A	10 ppm	20.11 B
**AtTa0112**	18.41 B	100 ppm	15.55 C
**AtAc0114**	15.66 C	1,000 ppm	9.66 D
**LSD value**	0.462	LSD value	0.533

** = significant at 5% level; ns = not significant

*Effect of methanol extracts of Chinigura rice grain on crown-gall tumors produced by A. tumefaciens on potato discs*

**Figure 7:**
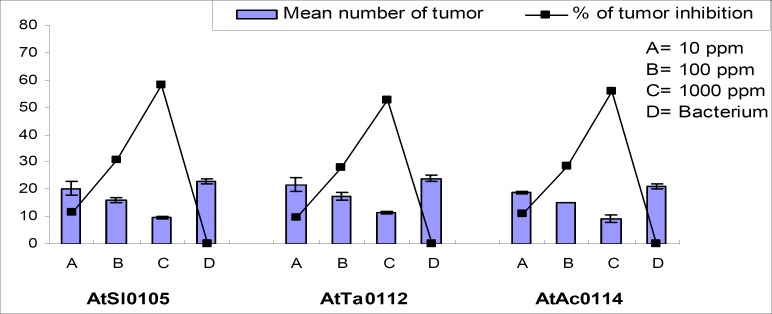
Effect of methanol extract of Chinigura rice grain on crown-gall tumor production by *A. tumefaciens *on potato discs.

**Figure 8:**
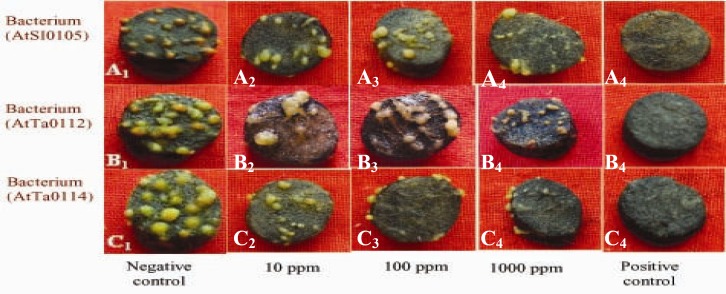
Photographs showing gradual tumor inhibition by the methanol extract of Chinigura rice grain on potato discs in a concentration as well as strain dependent manner. Data were compared with control. A_1_, B_1_, and C_1_ as negative control and A_2_, B_2_, and C_2_ as 10 ppm, A_3_, B_3_, and C_3 _as 100 ppm, A_4_, B_4_, and C_4_ as 1,000 ppm plant extract and A_5_, B_5_, and C_5_ as 30 ppm Camptothecin (positive control).

Significant tumor inhibition was observed at 10, 100, and 1,000 ppm Chinigura rice grain extracts compared with the negative control in all the strains (Table 4). Maximum 57.97%, 52.78%, and 55.56 and minimum, 11.59%, 9.72%, and 11.11% tumor inhibition were recorded for AtSl0105, AtTa0112, and AtAc0114 *Agrobacterium *strains, respectively (Figures 7 and 8). It was also observed that *A. tumefaciens *strain AtTa0112 was more prominent for producing tumor (18.53±2.78) followed by strains AtSl0105 (17.25±2.91), and AtAc0114 (16.00±2.54) (Table 4).

**Table 3:** Mean values for antitumor activity of methanol extract of Chinigura rice bran

**Variable**	**Mean number of tumor** **	**Variable**	**Mean number of tumors** **
**Strains**		Negative control	22.66 A
**AtSI0105**	15.16 A	10 ppm	18.55 B
**AtTa0112**	16.83 B	100 ppm	13.00 C
**AtAc0114**	14.33 C	1,000 ppm	7.55 D
**LSD value**	0.4.0	LSD value	0.466

** = significant at 5% level; ns = not significant

**Table 4:** Mean values for antitumor activity of methanol extract of Chinigura rice grain

**Variable**	**Mean number of tumor** **	**Variable**	**Mean number of tumors** **
**Strains**		Negative control	22.66 A
**AtSI0105**	17.25 A	10 ppm	20.22 B
**AtTa0112**	18.53 B	100 ppm	16.11 C
**AtAc0114**	16.00 C	1,000 ppm	10.11 D
**LSD value**	0.495	LSD value	0.572

** = significant at 5% level; ns = not significant

## Discussion

The present study reveals the effectiveness of aromatic rice genotypes (Kalijira and Chinigura) on inhibiting tumor formations. Results give a comparative overview on the effect of methanol extracts of bran and grain of Kalijira and Chinigura rice on crown-gall tumors produced by *A. tumefaciens* on potato discs. In our study, activity of methanol extracts increased with increasing concentration of the extracts indicating the efficiency as active antitumor agent. The maximum capacities of tumor inhibition by Kalizira and Chinigura rice bran were gradually increased at high concentrations (1,000 ppm). 

Takashima et al. (2011) ▶ also found similar results and stated that rice bran water extract (RBWE) and ethanol extract (RBEE) at 1.0 mgml^-1^ markedly inhibited the proliferation of LS174T human colon cancer cells. Barmes et al. (1983) ▶ and Verschoyle et al. (2007) ▶ also reported on antitumor activity of rice bran. Hayshi  et al. (1998) ▶ showed that two types of hydroxy acids, (10*E*, 12*Z*)-9-hydroxy-10,12-octadecadienoic acid and (9*Z*, 11*E*)-13-hydroxy-9,11-octadecadienoic acid, were obtained as cytotoxic compounds from a water extract of rice bran has prohibited activity against P388 mouse leukemia cells. Kannan et al. (2010) further isolated and fully characterized peptide derived from rice bran having anti-cancer properties. 

Results also revealed that Kalijira bran is more effective than Chinigura genotype. Kalizira rice bran is black/deep purple in color where pigmented bran is more useful and has antitumor properties. Nam et al. (2005) suggested that bran’s from pigmented rice varieties may provide a source of new natural antioxidants and anticarcinogens. Pigmented rice such as black, purple, or red rice is a good source of antioxidants as it contains anthocyanins that are effective free radical scavengers (Romero et al., 2009). Laokuldilok et al. (2011) ▶ reported that black rice bran contained gallic, hydroxybenzoic, and protocatechuic acids in higher contents than red rice bran and normal rice bran. Pigmented rice also contains anthocyanins. Anthocyanins are responsible for cyanic color of pigmented rice and are regarded as important nutraceuticals mainly due to their antioxidant effect, which provide a potential to prevent various diseases associated with oxidative stress (Duthie et al., 2000 ▶; Kong et al., 2003 ▶) what is related with cancer.

In our study, both rice grains showed comparatively higher antitumor properties (Kalizira 57.43% and Chinigura 55.53%). Hudson et al. (2000) ▶ also investigated potential colon and breast tumor-suppressive properties of rice, testing the hypothesis that rice contains phenols that interfere with the proliferation or colony-forming ability of breast or colon cells. Furihata et al. (1996) stated that rice extract prevented the damage and reduced the increase in replicative DNA synthesis. Fang et al. (2004) ▶ suggests that the pure rice phytochemicals that are anti-carcinogenic are more potent. Huia et al. (2010) ▶ reported that black rice extract appears to have anticancer effects against human breast cancer cells by inducing apoptosis and suppressing angiogenesis. Moreover, Muraguchi et al. (2011) examined polished rice as natural sources of cancer-preventing geranylgeranoic acid.

Between bran and grain, bran showed higher antitumor activity although it is an underutilized co-product from rice milling and generally used as animal feed. However, now it has long been considered as an excellent source of vitamins and other nutrients. Rice bran is a good source of antioxidants including vitamin E and oryzanol, high quality oil and protein, and cholesterol-lowering waxes and anti-tumor compounds such as rice bran saccharide (Takeshita et al., 1992 ▶; Qureshi et al., 2000 ▶). 

It can be concluded that two parts (bran and unpolished grain) of studied genotypes Kalijira and Chinigura have significant antitumor properties where pigmented Kalijira bran was prominent to have antitumor activity compared with the others. In this situation, if we could discover more antitumor compounds containing rice genotypes and increase the level of those compounds in our daily diet rice, e.g., golden rice or beta-carotene containing rice, it would be beneficial.
